# Siglec-E Promotes β2-Integrin-dependent NADPH Oxidase Activation to Suppress Neutrophil Recruitment to the Lung*

**DOI:** 10.1074/jbc.M114.574624

**Published:** 2014-06-03

**Authors:** Sarah J. McMillan, Ritu S. Sharma, Hannah E. Richards, Vikas Hegde, Paul R. Crocker

**Affiliations:** From the ‡ Department of Cell Signalling and Immunology, College of Life Sciences, and; § Centre for Dermatology and Genetic Medicine, Division of Molecular Medicine, University of Dundee, Dundee DD1 5EH, Scotland, United Kingdom

**Keywords:** Lipopolysaccharide (LPS), Lung Injury, NADPH Oxidase, Neutrophil, Reactive Oxygen Species (ROS), Siglec-E

## Abstract

Siglec-E is a sialic acid-binding Ig-like lectin expressed on murine myeloid cells. It has recently been shown to function as a negative regulator of β2-integrin-dependent neutrophil recruitment to the lung following exposure to lipopolysaccharide (LPS). Here, we demonstrate that siglec-E promoted neutrophil production of reactive oxygen species (ROS) following CD11b β2-integrin ligation with fibrinogen in a sialic acid-dependent manner, but it had no effect on ROS triggered by a variety of other stimulants. Siglec-E promotion of ROS was likely mediated via Akt activation, because siglec-E-deficient neutrophils plated on fibrinogen exhibited reduced phosphorylation of Akt, and the Akt inhibitor, MK2206, blocked fibrinogen-induced ROS. *In vivo* imaging showed that siglec-E also promoted ROS in acutely inflamed lungs following exposure of mice to LPS. Importantly, siglec-E-promoted ROS were required for its inhibitory function, as the NADPH oxidase inhibitor, apocynin, reversed the siglec-E-mediated suppression of neutrophil recruitment and blocked neutrophil ROS production *in vitro*. Taken together, these results demonstrate that siglec-E functions as an inhibitory receptor of neutrophils via positive regulation of NADPH oxidase activation and ROS production. Our findings have implications for the inhibitory role of siglec-9 on human neutrophils in sepsis and acute lung injury.

## Teaser

**Background:** Siglec-E is a negative regulator of neutrophil recruitment to the lung in a mouse model of sepsis.

**Results:** Siglec-E promotes integrin-dependent production of reactive oxygen species (ROS) and Akt activation.

**Conclusion:** Siglec-E-promoted ROS plays a key role in its suppression of neutrophil recruitment to the lung.

**Significance:** Siglecs may be new therapeutic targets in inflammatory lung disease.

## Introduction

Neutrophils are the most abundant blood leukocyte and play crucial roles in host defense to bacterial and fungal pathogens. Regulation of their entry into tissues from blood is essential for preventing bystander damage. This is particularly important in the lung, because there is a vast network of narrow alveolar capillaries that results in the margination of up to 60% of the circulating pool of neutrophils (1). These slowly crawling lung neutrophils are poised to respond to pathogenic threats via up-regulation of adhesion receptors and activation of integrins, leading to firm arrest and transmigration into the neighboring tissues via chemokine gradients. If these events are insufficiently controlled, it can lead to acute lung injury and the acute respiratory distress syndrome (2, 3).

In a murine model of sepsis, we recently identified siglec-E as an important negative regulator of β2-integrin-dependent neutrophil recruitment to the lung following exposure to lipopolysaccharide (LPS) (4). This was associated with a siglec-E-dependent reduction in phosphorylation of Syk-Tyr^317^ and p38 MAPK in neutrophils activated via β2-integrin ligation to fibrinogen (4). Although it is plausible that these pathways are involved in suppression of neutrophil recruitment *in vivo*, another important Syk-dependent pathway triggered by β2-integrins is activation of the NADPH oxidase complex and production of reactive oxygen species (ROS).^3^ This complex can be assembled and activated in response to a wide variety of stimulants, and it is known to play a critical role in antimicrobial host defense as revealed by genetic deficiencies of NADPH oxidase in humans (5) and mice (6). It has also become clear from studies of NADPH oxidase-deficient mice that phagocyte-derived ROS mediate an important anti-inflammatory function in the lung, leading to suppression of neutrophil recruitment (4, 7–9). Here, we demonstrate that siglec-E is important for promoting β2-integrin-dependent ROS production by neutrophils and that this pathway is required for siglec-E-dependent suppression of neutrophil recruitment to the lung.

## EXPERIMENTAL PROCEDURES

### 

#### 

##### Mice

Generation of siglec-E^R126D^ mice with a “knock-in” mutation in the sialic acid-binding site has previously been described (4). To generate a complete knock-out of siglec-E on a C57BL/6 background, we further crossed siglec-E^R126D^ mice with transgenic (Nes-cre)1Wme/J (Bal1 cre) mice to partially deplete the loxP-flanked allele. Mosaic offspring were then used to generate mice homozygous for the KO allele, which was confirmed by PCR. The following primers gave rise to a wild-type product (260 bp) and a KO product (394 bp): SigE KI 1524_39, CAGCAAAGCCATGGAGTTCC; SigE KI 1524_40, CACAATGTAGCAAGGATGACC; and SigE KI 4221_42, TAAATGCCAGCTGGATATGGTG. All mice were bred and maintained under specific pathogen-free conditions at the University of Dundee. All procedures were carried out with institutional ethics approval, under home office license, and were performed in accordance with the United Kingdom 1986 Animals (Scientific Procedures) Act. Lack of siglec-E expression was confirmed by flow cytometry of neutrophils from siglec-E KO mice using specific polyclonal antibodies.

##### In Vivo Models

Groups of sex- and age-matched WT and siglec-E KO mice were exposed to aerosolized LPS as described (4). For *in vivo* bioimaging of ROS, mice were injected intravenously with 25 mg/kg L-012 (WAKO) 3 h post-LPS. Mice were immediately anesthetized (Isofluorane), and dissected lungs were bioimaged using Xenogen IVIS-200 imaging system (PerkinElmer Life Sciences) from 5 to 10 min post-injection of L-012 (10). The resulting light emission was quantified using LivingImage software 3.0 (PerkinElmer Life Sciences). To investigate the effects of blocking NADPH oxidase *in vivo*, 20 μg of apocynin (Abcam) was instilled intranasally, 30 min before and 30 min after LPS. 3 h later, lung tissue was collagenase-digested and processed for cellular analysis as described (4).

##### Chemiluminescent Detection of ROS

ROS production was measured using a luminol-based assay in 96-well plates as described (11, 12). Briefly, bone marrow cells (6.25 × 10^6^/ml) were primed with or without TNF-α (20 ng/ml, PeproTech) for 10 min at 37 °C. Where indicated, cells were preincubated with the Akt inhibitor, MK2206 (Selleckchem), or NADPH oxidase inhibitor, apocynin or vehicle control (DMSO, 0.1%), for 20 min prior to measurement. Cells were incubated with luminol (150 μm, Sigma) and HRP (18.75 units/ml, Sigma) and added to wells precoated with fibrinogen (100 μg/ml, Sigma), poly-RGD (20 μg/ml, Sigma), or immune complexes (12) or that contained PDBu (100 nm, Sigma), serum-opsonized zymosan (100 μg/ml opsonized at a ratio of 1:1 in 10% normal mouse serum for 1 h 37 °C, Invivogen), LPS (10 μg/ml, Sigma), or *Staphylococcus aureus* (1:50 ratio of cells/bacteria), and measurement was started immediately. In some experiments, fibrinogen and poly-RGD were treated with sialidase from *Vibrio cholerae* (Sigma) in sodium acetate buffer, pH 5, containing 2 mm CaCl_2_ for 1 h at 37 °C prior to plating the cells. Light emission was recorded every minute for 1 h by a FLUOstar Optima (BMG Labtech).

##### Biochemical Analysis

Biochemical analysis of bone marrow cells plated on fibrinogen was performed as described previously (4). Lysates containing equal amounts of proteins were subjected to immunoblotting with antibodies against total and phospho-Akt (Thr-308 and Ser-473, Cell Signaling).

##### Data Analysis

Data are expressed as means ± S.D. for *in vitro* assays or means ± S.E. for *in vivo* experiments. Statistical significance between groups was tested using a Mann Whitney *U* test. A *p* value of equal to or less than 0.05 was considered significant.

## RESULTS

### 

#### 

##### Selective Defect in β2-Integrin-dependent ROS Production in Siglec-E-KO Mice

**FIGURE 1.**
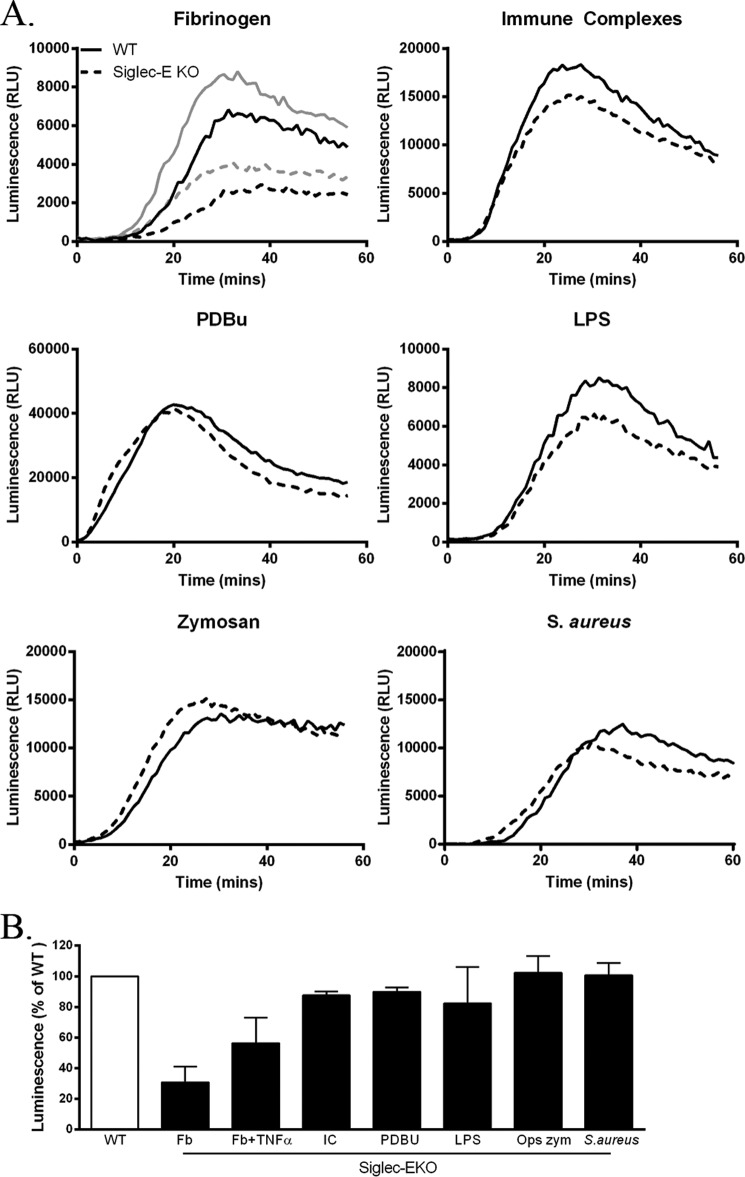
**Selective defect in β2-integrin-dependent ROS in siglec-E-KO mice.** *A,* ROS-dependent chemiluminescence shown as relative light units (*RLU*) using bone marrow cells from WT and siglec-E KO mice stimulated as indicated. *Solid traces,* WT; *dashed traces*, siglec-E KO; *gray traces* in the fibrinogen (*Fb*)-treated group indicate TNF-α priming. *B,* luminescence responses in siglec-E KO cells expressed as a percentage of values seen with WT cells; mean ± S.D. from triplicate wells from at least two independent experiments. *IC*, immune complex; *OPS zym*, serum-opsonized zymosan.

To investigate whether siglec-E is important for regulating neutrophil ROS production, we compared responses of WT and siglec-E KO bone marrow neutrophils using a well established luminol-based chemiluminescence assay (11, 12) with a range of stimulants (Fig. 1). Siglec-E KO neutrophils showed a clear defect in β2-integrin-triggered ROS production when cells were plated on fibrinogen, which was apparent in the absence or presence of TNF-α priming (Fig. 1). The effect was selective to fibrinogen-mediated β2-integrin signaling because normal ROS responses were observed with siglec-E KO neutrophils using immune complexes, the phorbol ester PDBu, LPS, serum-opsonized zymosan, and *S. aureus* (Fig. 1).

##### Siglec-E-dependent Promotion of β2-Integrin-triggered ROS Requires Sialic Acid Recognition

**FIGURE 2.**
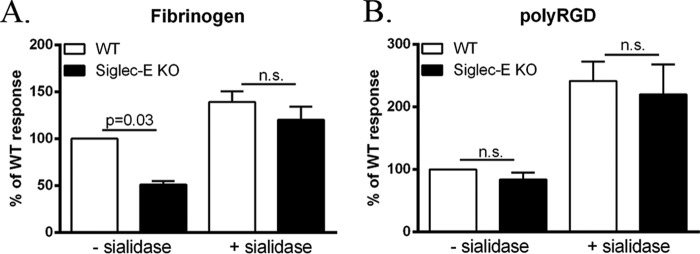
**Siglec-E-dependent activation of β2-integrin-triggered ROS production requires sialic acid recognition.** *A,* luminescence responses of TNF-α-primed WT and siglec-E KO cells plated onto fibrinogen (*A*) or unprimed cells plated onto poly-RGD (*B*)-coated wells both pretreated with or without sialidase in sodium acetate buffer, pH 5. Luminescence responses are expressed as the percentage of TNF-α-primed WT cells plated onto buffer-treated fibrinogen (*A*) or WT response to buffer-treated poly-RGD (*B*). Data show means ± S.D. from triplicate wells from seven independent experiments.

We have previously shown that sialic acid recognition of the β2-integrin ligand, fibrinogen, by siglec-E is required for inhibitory signaling (4). To investigate whether this was also the case for siglec-E-dependent ROS production, we compared responses of WT and siglec-E KO cells plated either on fibrinogen, with or without sialidase treatment, or on the nonglycosylated β2-integrin ligand, poly-RGD (13). Fig. 2*A* shows that the siglec-E-dependent promotion of ROS was lost following sialidase pretreatment of fibrinogen. Furthermore, comparable β2-integrin-triggered ROS production was induced by poly-RGD in WT and siglec-E KO cells (Fig. 2*B*). For unknown reasons, sialidase treatment led to a nonspecific increase in ROS production in all groups tested (Fig. 2).

##### β2-Integrin-triggered Akt Activation Is Promoted by Siglec-E and Is Required for ROS Production

**FIGURE 3.**
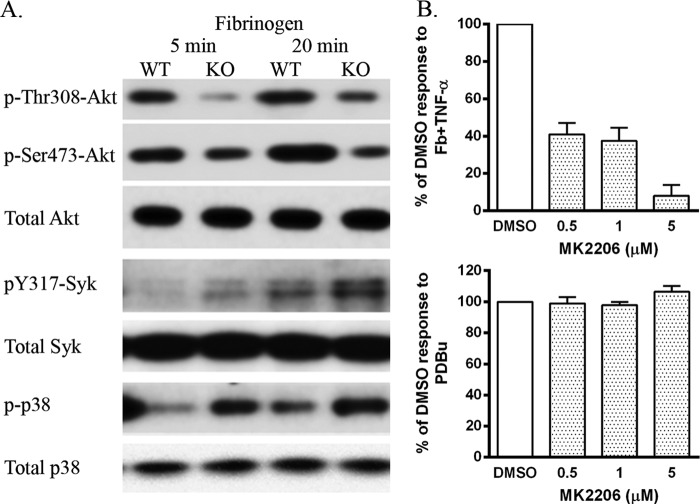
**β2-Integrin-triggered Akt activation is promoted by siglec-E and is required for ROS production.** *A,* Western blots were prepared from WT and siglec-E KO bone marrow cells plated on wells precoated with fibrinogen for 5 or 20 min at 37 °C and probed with the indicated antibodies. Data are representative of two independent experiments. *B,* ROS-dependent chemiluminescence using bone marrow cells from WT mice pretreated with the Akt inhibitor, MK2206, or DMSO as a control. Luminescence responses are expressed as the percentage of DMSO-treated controls from MK2206-treated, TNF-α-primed cells plated onto fibrinogen (*Fb*) or stimulated with PDBu. Data show means ± S.D. from triplicate wells from three to four independent experiments.

In view of the importance of PI3K activation in triggering ROS production in neutrophils (11), we analyzed Akt Ser^473^ and Thr^308^ phosphorylation as readouts of phosphatidylinositol 1,4,5-trisphosphate levels (Fig. 3*A*) (14). Phosphorylation of both Akt sites was reduced in siglec-E KO cells at both 5 and 20 min following plating on fibrinogen (Fig. 3*A*). This was in contrast to increased phosphorylation of Tyr^317^-Syk and p38 MAPK seen in siglec-E KO neutrophils (Fig. 3*A*) and reported previously (4). To investigate whether Akt was required for β2-integrin-dependent ROS production in WT neutrophils, we used the specific Akt inhibitor MK2206 (15) and observed a dose-dependent reduction of ROS in response to fibrinogen (Fig. 3*B*, *upper plot*), but there was no effect on the response to PDBu (Fig. 3*B*, *lower plot*). Therefore, these data suggest that siglec-E promotes β2-integrin-dependent ROS production via an Akt-dependent pathway.

##### Siglec-E-dependent Production of Neutrophil ROS in Lungs of LPS-exposed Mice

**FIGURE 4.**
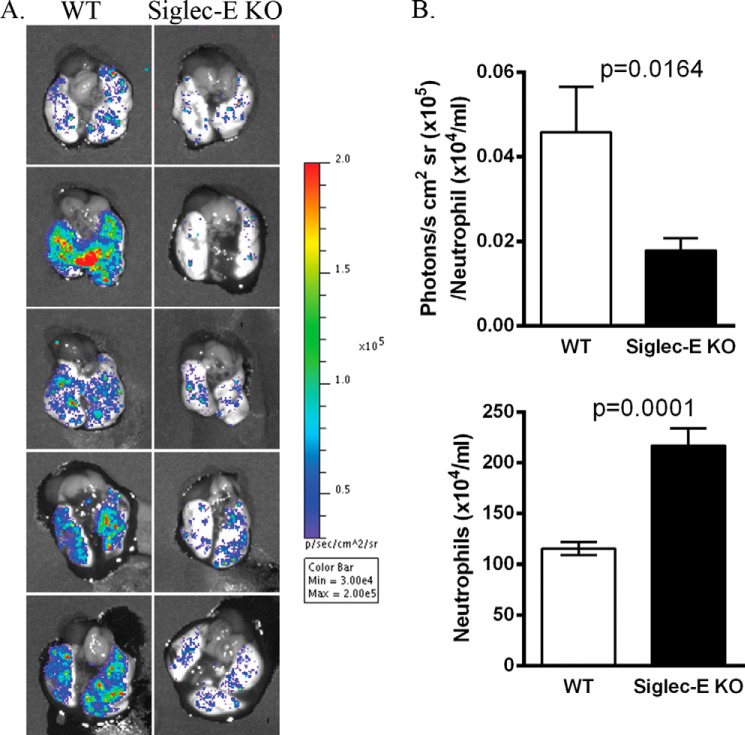
**Siglec-E-dependent production of neutrophil ROS in lungs of LPS-exposed mice.** Groups of mice were exposed to aerosolized LPS. 3 h later, mice were injected intravenously with L-012 and anesthetized, and excised lungs were bioimaged from 5 to 10 min. *A,* chemiluminescent signals from representative lungs of five mice per genotype. *Color bar* depicts luminescent light emission intensity (photons/s/cm^2^/sr). *B, upper panel* shows luminescence signals normalized to neutrophil counts obtained from collagenase-digested lung (*lower panel*). Data are expressed as means ± S.E., *n* = 14 per group. *p* values were calculated using the Mann Whitney *U* test.

To determine whether siglec-E KO neutrophils exhibited defective ROS production *in vivo*, we utilized a bioimaging technique in which mice were exposed to aerosolized LPS for 3 h and then injected with the chemiluminescent probe L-012 (10) prior to removing dissected lungs for imaging (Fig. 4). After normalizing for neutrophil number, the ROS signals in siglec-E KO mice were significantly reduced in comparison with WT mice, concomitant with increased neutrophil numbers (Fig. 4) as reported previously (4). Therefore, siglec-E positively regulates ROS production by neutrophils both *in vitro* and *in vivo*.

##### Blockade of NADPH Oxidase in Vivo Reverses Siglec-E-dependent Suppression of Neutrophil Recruitment

**FIGURE 5.**
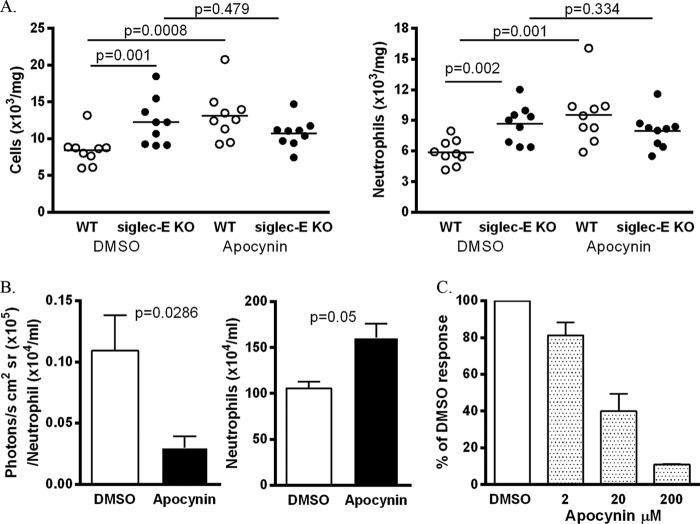
**Blockade of NADPH oxidase *in vivo* reverses siglec-E-dependent suppression of neutrophil recruitment.** *A,* mice were pretreated with apocynin or DMSO vehicle as control and then exposed to aerosolized LPS. After 3 h, lung tissue was collagenase-digested, and total cells and neutrophils were enumerated. Data are expressed as scatterplots with the *bars* depicting the means, *n* = 9 per group from two independent experiments. *p* values were calculated using the Mann Whitney *U* test. *B,* WT mice were treated as in *A. Left panel* shows luminescence signals from *in vivo* bioimaging after normalizing for neutrophil counts obtained from collagenase-digested lung tissue (*right panel*). Data are expressed as means ± S.E., *n* = 4 per group. *p* values were calculated using the Mann Whitney *U* test. *C,* bone marrow cells were pretreated with the indicated concentrations of apocynin or with DMSO vehicle control for 20 min, and luminescence was measured using wells precoated with fibrinogen. Data are presented as means ± S.D. representative accumulated light emission (relative light units (*RLU*))over time from triplicate wells from duplicate samples.

To investigate a potential link between siglec-E-dependent neutrophil ROS production and suppression of neutrophil recruitment to the lung, we compared the LPS-induced cellular responses of WT and siglec-E KO mice following treatment with the NADPH oxidase inhibitor, apocynin (10). Interestingly, both total lung cell numbers and neutrophils were significantly increased in apocynin-treated WT mice (Fig. 5*A*), whereas this treatment had no effect on siglec-E KO mice in comparison with DMSO controls (Fig. 5*A*). Following apocynin treatment, there was no longer any difference in neutrophil numbers in the lungs, comparing WT and siglec-E KO mice (Fig. 5*A*; *p* = 0.16). As expected (10), apocynin blocked neutrophil ROS production in lungs of mice exposed to LPS (Fig. 5*B*) and inhibited β2-integrin-dependent ROS production in a dose-dependent manner (Fig. 5*C*).

## DISCUSSION

The major conclusion of this study is that the inhibitory receptor, siglec-E, suppresses neutrophil recruitment to the inflamed lung via stimulation of ROS production. This was supported by the following: (i) siglec-E promoted β2 integrin-dependent ROS release by neutrophils, both *in vitro* and *in vivo,* and (ii) apocynin, an inhibitor of NADPH oxidase assembly, reversed the siglec-E-dependent suppression of neutrophil recruitment to the lungs of mice exposed to LPS. Our finding that siglec-E-dependent production of ROS in the lung is an important anti-inflammatory mechanism is consistent with a series of studies demonstrating exaggerated inflammatory responses in mice lacking the p47phox subunit of the NADPH oxidase complex (7, 8, 16). It was recently shown that local production of ROS in response to intratracheal LPS instillation resulted in decreased NF-κB DNA binding activity due an altered redox state in macrophages (4). Superoxide anions and hydrogen peroxide can enter other cells via chloride channels and aquaporins, respectively (17), so it is possible that siglec-E-dependent production of neutrophil ROS results in reduced transcriptional activity of NF-κB in macrophages, resulting in decreased neutrophil recruitment into the lung.

The mechanism by which siglec-E promotes β2-integrin-dependent ROS production is unclear, but it is likely to involve PI3K activation, generation of phosphatidylinositol 1,4,5-trisphosphate, and Akt activation because siglec-E-deficient neutrophils showed reduced levels of Akt phosphorylation following exposure to fibrinogen, and ROS production was blocked by the Akt-specific inhibitor MK2206. Previous work showed that the Akt2 isoform is important for neutrophil ROS production and that Akt is required for p47^phox^ phosphorylation and NADPH oxidase activation (18–20). The finding that siglec-E positively regulates β2-integrin-dependent phosphorylation of Akt contrasts with our previous observation that siglec-E negatively regulates phosphorylation of Syk-Tyr^317^ and p38 MAPK. Although Syk protein is required for integrin-dependent ROS production (13), the importance of its catalytic activity has been questioned (21). It seems likely that siglec-E regulates at least two signaling pathways downstream of β2-integrin ligation in neutrophils, one leading to suppression of Syk-Tyr^317^ phosphorylation and p38 MAPK activation, and the other involving Akt activation and ROS production. We propose that siglec-E-dependent modulation of these two β2-integrin signaling pathways constitutes a coordinated response resulting in anti-inflammatory signaling and protection of the lung against neutrophil injury.

Using fibrinogen as a naturally sialylated β2-integrin ligand, we showed previously that sialic acid-dependent co-recognition by siglec-E was important for its inhibitory signaling function (4). Similarly, we showed here that sialic acids on fibrinogen were required for siglec-E-dependent promotion of ROS. Taken together, these findings indicate that the siglec-E-dependent modulation of β2-integrin signaling requires *trans* interactions between siglec-E on neutrophils and sialic acids presented on β2-integrin ligands or other associated molecules. This may be important for bringing siglec-E and β2-integrin in sufficient proximity to permit siglec-E-dependent functional responses. These signaling events are likely to occur during the initial interactions between neutrophils and lung microvascular endothelium following exposure to LPS (1, 2, 4).

Our *in vitro* studies clearly showed that although siglec-E promoted ROS production of neutrophils in response to fibrinogen, it did not influence ROS production using a range of other stimulants, including LPS, serum-opsonized zymosan, and *S. aureus*. Because these stimulants are thought to be recognized by other pathogen recognition receptors as well as CD11b β2-integrin (22), siglec-E is unlikely to influence ROS production when neutrophils encounter nonsialylated pathogens and pathogen products. It is well established that neutrophil ROS are crucial for host defense functions and pathogen killing. In comparison, siglec-E-dependent ROS produced via recognition of “self”-components such as fibrinogen may play a regulatory role in controlling neutrophil recruitment to the inflammatory site and hence limiting bystander tissue damage.

In conclusion, we demonstrate that siglec-E is able to positively regulate Akt-dependent ROS production downstream of β2-integrin ligation both *in vitro* and *in vivo* and is required for siglec-E-dependent suppression of neutrophil recruitment to the lung in response to LPS. Our findings have important implications for the functions of the highly related human siglec-9 expressed by neutrophils (23, 24) and raise the possibility that siglecs could be new therapeutic targets in neutrophil-driven inflammatory lung disease.

## Footnotes

3 The abbreviations used are:

ROS: reactive oxygen species
siglec: sialic acid-binding immunoglobulin-like lectin
PDBu: phorbol 12,13-dibutyrate.
